# Examining missing pieces of the human resource (HR) attributions puzzle: The interplay between line manager beliefs, HR information and context

**DOI:** 10.3389/fpsyg.2023.1103996

**Published:** 2023-02-16

**Authors:** Hertta Vuorenmaa, Jennie Sumelius, Karin Sanders

**Affiliations:** ^1^School of Business, Aalto University, Espoo, Finland; ^2^Hanken School of Economics, Helsinki, Finland; ^3^Stockholm School of Economics, Stockholm, Sweden; ^4^School of Management and Governance, Business School, University of New South Wales, Sydney, NSW, Australia

**Keywords:** HR process, line managers, HR department, HR attributions, context

## Abstract

While previous research acknowledges the importance of line manager interpretations of information coming from the HR department for explaining various employee attitudes and behaviors, less is known about the antecedents of these interpretations, also known as HR attributions. This paper provides a qualitative examination of the interplay between three key antecedents of HR attributions, namely, line manager *beliefs* about the HR department, *information* from the HR department and *context.* Our analysis is based on 30 interviews with HR professionals and line managers in three units of one organization. Our findings suggest that differences in context have a strong impact on line manager beliefs about HR, influencing the way line managers see HR practices, processes and the role of the HR department, and consequently the way they interpret information coming from HR. Our analysis extends our understanding of the variability in line manager interpretations of HR information. Our results contribute to existing research on HRM strength and HR attributions by highlighting the importance of focusing not only on the consistency of the HR system, but also on individual line managers beliefs about HR, and the context in which HR processes take place.

## Introduction

Attribution theories (Heider, 1958; Kelley, 1967, 1973; Weiner, 1972) assume that people act as naïve psychologists, developing explanations for their own actions, experiences, observed events, or social encounters both in life in general, and at work. The central tenet underlying existing research on attributions is that *“people interpret behavior in terms of its causes and that these interpretations play an important role in determining reactions to the behavior”* (Kelley and Michela, 1980: 458). In addition, these attributions enhance people’s ability to understand, predict, and control their environment, especially in times of uncertainty (Wong and Weiner, 1981). Previous research in the human resource (HR) field has found support for the effect of employees’ (positive and negative) attributions about HR practices on various employee outcomes such as satisfaction and commitment (Nishii et al., 2008; Van De Voorde and Beijer, 2015; Sanders et al., 2021a), task performance (Chen and Wang, 2014), affective commitment (Fontinha et al., 2012), and emotional exhaustion (Shantz et al., 2016; see also Hewett, 2021 for an overview).

Although existing research on HR attributions has provided valuable insights into the role that attributions about HR practices play in influencing employee attitudes and behaviors, we know less about how these attributions are formed (Hewett et al., 2018; Hewett, 2021; Sanders, 2022) and what factors lead to variability in HR attributions (Van Rossenberg, 2021), since *“most scholars view variability in HR attributions as a measurement error that needs to be reduced* (Van Rossenberg, 2021), *rather than as a variable that can be purposefully studied”* (Meier-Barthold et al., 2022:2). If attributions are argued to influence subsequent employee attitudes and behaviors, it is important to deepen our understanding of the elements that contribute toward shaping them. Kelley and Michela (1980) suggest that there are three key factors shaping attributions, namely, information, beliefs, and motivation and call for more research specifically into the interplay between these processes. Hewett et al. (2019) applied Kelley and Michela’s (1980) principles of information (perceptions of distributive and procedural fairness), beliefs (organizational cynicism), and motivation (perceived relevance) as the antecedents of attributions regarding the purpose of a very specific HR practice, namely, the workload management framework. Their quantitative study among 347 academic faculty in the UK shows that fairness (information) and cynicism (beliefs) interact so that perceived fairness buffers the negative effect of cynicism.

This paper builds on the findings of Hewett et al. (2019), as well as additional research on the antecedents of HR attributions (Van De Voorde and Beijer, 2015; Alfes et al., 2021; Guest et al., 2021; Sanders et al., 2021b) and variability on employee attributions (Meier-Barthold et al., 2022), this paper examines the interplay between information, beliefs and motivation in the context of three different units of one organization. Specifically, we examine how line managers’ beliefs regarding the HR department are shaped by the information they receive from HR as well as their motivation conceptualized as unit context. The lack of more focused attention on line manager beliefs about HR is surprising given the importance of them internalizing the value of HR in order to devote sufficient time and attention to people issues (Björkman et al., 2011).

With this study we contribute to existing HR literature in three ways. First, and most importantly, through our in-depth qualitative examination based on 30 interviews with HR and line managers in three units all belonging to one Nordic IT company, we shed light on the *variability of line manager beliefs of HR within organizations* (Nishii and Wright, 2008; Wright and Nishii, 2013). We illustrate how line manager beliefs of HR vary despite the same message (information) from the corporate HR department, in particular our findings highlight the importance of the role of the unit context in which the line managers are nested (Kelley, 1967, 1973; Cooke, 2018). In this way, we build on existing theoretical and empirical work on HRM system strength (Bowen and Ostroff, 2004; see also Ostroff and Bowen, 2016; Sanders, 2022) to further explain the information element. The HRM system strength line of research assumes that if an HRM systems strength is characterized as high distinctiveness, high consistency and high consensus, it produces similar expectations and behaviors among employees. Our findings show that in order to understand the effects and strength of the HR system (information from the HR department), it is crucial to also understand the beliefs of the line managers in that given HR system and the context in which the HR processes take place. Our research contributes to research on HRM system strength by elaborating on the elements that shape employee HR attributions and furthering our understanding of the microprocesses through which HRM influences performance (Hewett et al., 2018; Hewett, 2021).

Second, moving away from a purely universalistic perspective of HRM system strength, our study contributes by incorporating a focus on the boundary conditions of HRM system strength. Kelley and Michela (1980) specifically called for more research on the motivation element, as it has been less examined as compared to the information and beliefs elements. We show that contextual unit differences play an important role in line managers’ motivation to make sense of the information from the HR department as intended. Our study thus contributes by responding to calls to reintroduce the role of context into the HR field in order to better make sense of *how* and *why* HRM unfolds the way it does within organizations (Cooke, 2018).

Third, while previous research on the antecedents of attributions primarily focuses on the individual level (Kelley and Michela, 1980; Hewett et al., 2019), our approach includes antecedents at individual (line manager beliefs), organizational (information from the corporate HR department) and unit levels (unit context). Building on previous research (e.g., Lin and Sanders, 2017; see also Rabl et al., 2014; Wang et al., 2020) which has shown that behaviors are indeed influenced by an interplay of antecedents at different levels, we argue that it is important to acknowledge how the complex interplay of these antecedents at different levels leads to variability of HRM, and also affects the way in which HR information is received.

## Theoretical background

Elements of attribution theories have previously been applied in influential streams of HR research. To date HR research on *causal attribution theory* has mainly focused on the influence of employee perceptions about the organizational intentions behind the implementation of HR practices i.e., HR attributions (Nishii et al., 2008). Subsequent research in this area (e.g., Fontinha et al., 2012; Van De Voorde and Beijer, 2015; Sanders et al., 2021a; see also Hewett, 2021, and Sanders, 2022 for overviews) shows that when employees believe the HR practices within their organization have been implemented to enhance employee wellbeing or service quality (so called positive HR attributions), they tend to report higher organizational commitment and are more satisfied in comparison to employees who believe that HR practices are designed to intensify work and/or reduce costs (so called negative HR attributions).

Another line of research on attributions and HR by Bowen and Ostroff (2004), see also Ostroff and Bowen (2016), focuses on employees’ understanding of HR practices. Drawing on Kelley’s *co-variation model of attribution theory* (Kelley, 1967, 1973), Bowen and Ostroff argued that HR practices represent signals to employees, sent out by management. If these HR messages are perceived by employees as distinctive (HR practices stand out in comparison to other messages from the organization), consistent (HR practices send out a similar signal), and consensual (HR practices are perceived in the same way), this should result in a “strong” HRM system in which employees understand what is expected from them and what is in turn rewarded (see also Sanders and Yang, 2016).

In their seminal review article, Kelley and Michela (1980) connected these approaches, and argued that the way in which employees receive and understand information (*the co-variation model of the attribution theory*) is one of the antecedents of how people make causal attributions (*causal model of attribution theory*). Thus, in addition to the importance of distinctive, consistent, and consensual *information* for shaping an individual’s causal attributions, Kelley and Michela (1980) argued for the importance of *beliefs* and *motivation* of individuals (Bennett, 2017; Perry and Hamm, 2017) as additional factors influencing the formation of attributions. While research in the area of HR attributions has focused extensively on examining various outcomes, Hewett et al. (2018) and Hewett et al. (2019) recently argued that this focus is too restricted, and that in order to increase our understanding of the microprocesses through which HR affects performance, we need to learn more about different antecedents of attributions. This paper thus provides a qualitative examination of the interplay between *information* from the HR department, line manager *beliefs* about the HR department, and the unit context (*motivation*).

### Information

According to the co-variation model of attribution (Kelley, 1967, 1973), individuals make confident attributions about cause-effect relationships depending on the degree of distinctiveness (the event-effect is highly observable), consistency (the event-effect presents itself the same across modalities and time) and consensus (there is agreement among individual views of the event-effect relationship). Building on this, the concept of *HRM system strength* (Bowen and Ostroff, 2004; Ostroff and Bowen, 2016), suggests that HR practices can be viewed as a message-based persuasion process from employers to employees that is aimed at influencing employee attitudes and behaviors (Guzzo and Noonan, 1994; Bowen and Ostroff, 2004). In the context of this paper, we conceptualize *information* in line with Bowen and Ostroff’s (2004) HRM system strength framework, focusing on the distinctiveness, consistency, and consensus of the information sent out by the HR department.

### Beliefs

Heider (1958) argued that people’s attributions of the actions of others are informed by their *general beliefs* about on-going experiences. Similarly, Kelley and Michela (1980) posited that people’s pre-existing suppositions and expectations about actors and their behavior in different situations commonly influences their processing of current information. In the context of this paper, we define general beliefs as the way in which line managers view the HR department. Based on their ongoing and previous experiences with HR, line managers harbor positive or negative beliefs about the HR department (Hu and Oh, 2022).

### Motivation

The final, and least researched element of Kelley and Michela’s (1980) framework is *motivation*. Motivation captures the extent to which the initiative in question is of interest to the attributor (Hewett et al., 2019). In our case these are the line managers in the different units. Drawing on social information processing theory (Salancik and Pfeffer, 1978), we argue that the way in which line managers understand and make sense of HR, is shaped by the informational and social context of the unit within which they perceive HR.

#### The interplay between information, beliefs, and motivation

Kelley and Michela (1980) elaborated on the relationship between information and beliefs, arguing that the way information is received can be greatly affected by peoples’ preconceptions about cause-effect relations. Kelley and Michela (1980) (p. 468) also mention that the variability regarding how information is received “*must be qualified in the light of the causal beliefs the attributor brings to most problems and his (sic!) varying motives related to achieving accurate understanding versus other end.”* The cognitive process through which individual beliefs are formed, however, is only undertaken if individuals believe the stimulus is significantly important to them (e.g., Petty and Cacioppo, 1986; Weiner, 1986).

**FIGURE 1:**
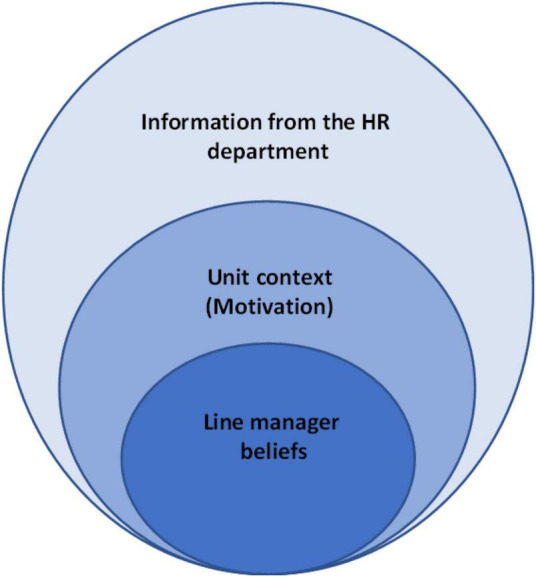
The relationship between the three key antecedents of HR attributions: Information, beliefs and context.

We suggest that a sole focus on HRM system strength features (distinctiveness, consistency and consensus; (Bowen and Ostroff, 2004; Ostroff and Bowen, 2016) is not sufficient, and that the interpretations and responses of the line managers, as well as the specific organizational context in which they operate, constitute important contextual elements of the environment that should be taken into account (Kuvaas et al., 2014; Meier-Barthold et al., 2022). Following Kelley and Michela (1980), we argue that line managers’ interpretation of the information they receive from the HR department contributes to shaping their beliefs about the HR department, its role and purpose, and that this is influenced by the context in which this takes place. Differences in unit context play an important role for line managers’ motivation to read the information from the HR department as it was intended and as such line manager beliefs (and motivation) about the HR department should be understood by considering the information from the HR department and the motivational context of the given unit. Our analysis contributes to existing research on HRM strength and HR attributions by examining the relationship between the consistency of the HR system, individual line managers beliefs about HR and the context in which HR processes take place (see Figure 1).

## Materials and methods

### The case context

Our case company (hereafter referred to as Tecco) is an IT company operating in the Nordic countries. It employs around 1,500 people primarily located in three units (CAPONE, CAPTOO, and NORDSIDE). CAPONE and CAPTOO are located in the metropolitan areas of two Nordic capital cities, whereas NORDSIDE is regionally located. Tecco has grown to its current size through several mergers and acquisitions over the years. The Tecco HR department (hereafter referred to as HR) consists of seven people working in three different locations. Despite its relatively small size, HR has earned external acclaim for its work in terms of two major national HR award. HR consists of a close-knit group of HR professionals taking great pride in their work and in cooperating well with each other. Together they are responsible for developing and maintaining the company’s HR processes and systems, and traditional HR practices such as recruitment and selection, compensation, training, and performance management. HR is the orchestrator of the company’s performance management process, although the implementation of it rests heavily on line managers. Training is one of HR’s core responsibilities, and HR is solely responsible for organizing the company’s bi-annual training/management day for line managers. In addition, HR is an active organizer of social events for all company staff, end of year parties and celebrations in conjunction with public holidays.

The three focal units; CAPTOO, NORDSIDE, and CAPONE vary in terms of age (time in the company), mode of establishment, the nature of the industry in which they operate etc., as illustrated in Table 1. CAPTOO operates in consulting and was acquired by Tecco 4 years ago. The work in NORDSIDE concentrates on customer service in a call center, coding, and providing various IT solutions for customers. This unit is the “cultural heart” of Tecco, still operating in the same place where the company was once founded. In CAPONE, work centers around providing tailor made digital services to varying business customers. This unit is the biggest out of the three, and also the one in which the main HR manager is physically located.

**TABLE 1:** Unit characteristics.

Contextual features	NORDSIDE	CAPONE	CAPTWO
Age	50 + years	15 years	4 years
Mode of establishment	Greenfield (original founding unit)	Acquisition	Acquisition
Nature of industry	Call center, coding and customer IT solutions and cloud servers	Coding, customer IT solutions, customer data servers and cloud solutions	Consultancy
Working culture	Strong hierarchy, low consensus seeking	Moderate hierarchy, expertise driven	Flat hierarchy, high consensus seeking, expertise driven
Location	Rural area	Metropolitan area	Metropolitan area

### Research approach and empirical material

Our approach to conducting fieldwork can best be described as open, emergent and explorative (Alvesson and Kärreman, 2007). We entered the field with the aim to learn more about how HRM unfolded in the organization, and our focus was on the work of the HR department. We interviewed members of the HR department about their everyday practices, what they view as most important goals in their job and how they go about getting their job done. We simultaneously interviewed other organizational members (top managers, line managers and employees) about their views of the HR department and how HR related to their own jobs. This study is based on 30 qualitative interviews conducted with line managers, as well as the HR manager and the other HR professionals working in the HR department.

We started the research process with quota sampling (Parker et al., 2019), where participants of the interviews were selected based upon being members of the company HR department. We started out by interviewing the HR manager (altogether three times). We explained that we sought to examine how HR was done in the company (how the HR department was structured, who did what HR-related tasks etc.) and that we were interested in gathering a variety of perspectives from different employee groups. The HR manager provided us with contact details to five line managers, as well as to all HR professionals in the HR department. In order to avoid the potential selection biases of the HR department, we continued the process of selecting interviewees by following the snowball sampling approach (Parker et al., 2019) so that at the end of each interview we asked participants for suggestions about other potential interviewees.

The interviews all followed a similar structure and a semi-structured interview *pro forma*, we had one *pro forma* for HR and another one for line and top managers. Rather than posing too many specific questions, we had some general themes we wanted respondents to talk about and then posed prompts, the purpose of which, as Jiménez and Orozco (2021:510) argue, is *“not to get the respondent to answer a specific question but rather to provide the respondent with a device to think through and discuss a set of topics.”* Our aim and interview technique were to let the interviewees talk as freely as possible (Seidman, 2019), and using prompts was a way of ensuring that the interviewees would cover the areas we were interested in while also trying not to avoid influencing their way of talking. The interviewees were thus first asked to tell us about more overarching themes such as their own work, their daily tasks and key responsibility areas. In our interviews with the HR professionals, we then focused primarily on what they do in their daily work in the HR department and why they do what they do. With line managers, after asking about their own work more generally, we then asked how HR relates to their own work, how they perceive HRM in the company and how they perceive the work and performance of the HR department, including its strengths and weaknesses, and what factors they saw as enabling and hindering good HR in their unit and in the organization in general. The interviews were all conducted face to face, by the two first authors as a pair, and always in the interviewee’s native language (Swedish or Finnish) in order to further enhance data quality. The interviews were all digitally recorded with the permission of the interviewees and professionally verbatim transcribed.

### Analysis

We adopted an abductive approach to the data analysis and analyzed the interviews through repeated iterations between theory and data as well as in reflexive discussions about our respective field notes. Our approach follows the ideas of Gephart (2004) and Pratt (2008) that “Qualitative research starts from and returns to words, talk, and texts as meaningful representations of concepts” (Gephart, 2004:455). We describe the phases of our analysis below, acknowledging the iterative nature of qualitative data analysis and the blurred boundaries between the phases (Barley et al., 2011). In the different phases we at times used the observation data in order to further contextualize the interview material.

*Step 1: Creating an understanding of the data.* The analysis for this paper started with the two authors that collected the data discussing the case, reading and re-reading the interview transcripts, looking for themes with the priority of letting the data speak. The aim at this stage was to develop an overall understanding of the story of the interview data. Although doing this independently, we regularly discussed emerging themes, respective views during the early, middle and later phases of data collection and exchanged and compared notes in order to further develop our understanding and test ideas, after which we then went back to analyzing the interviews. At this point we focused on letting emerging themes surface and re-surface (Van Maanen et al., 2007).

*Step 2: Raw analyses.* Next, we conducted raw analyses of the data, noting that the HR professionals talked a lot about their daily work, raising issues such spending a lot of time communicating to line managers about people related matters, and working to increase the internal and external visibility of HR. The managers also extensively discussed the need for consistency and alignment of HR practices across units (e.g., maintaining same policies and practices for everyone and the difficulty of this in repeated merger situations), and the importance of creating consensus about strategic and operational HR with top management. In the case of the line manager interviews, we already during this phase noted that the line managers appeared rather divided in how they interpreted HR signals.

*Step 3: Coding*. The HR professionals’ descriptions of how they went about their daily work and what they tried to achieve by doing things in the way they did fit with our initial thoughts of using the theory on HR process features, and we decided to start coding the data according to that. The third author joined the process at this stage without any prior involvement with the data, adding another layer to the analysis process in terms of approaching the data with new eyes and without the data collection bias of the two first authors. We started the coding process so that we divided the transcripts and all three authors identified excerpts in the interviews that related to distinctiveness, consistency and consensus, and line manager reactions to HR signals. We created a shared table with interview excerpts paired with comments about the issues and themes we found them to relate to (Strauss and Corbin, 1990). After this, we all coded the transcripts. In order to ensure the highest possible reliability and sense of context, we retained the original pieces of text used in our coding table (Bryman and Burgess, 2002), and regularly reflected, discussed and compared our notes and interpretations, and re-read all the interviews to revisit and double check our initial interpretations of the data. Early on we identified differences between line manager beliefs about the HR department and noticed that they seemed to depend on the unit in which they were located. This observation led us to focus on the unit context as the key factor influencing their interpretations and thus their motivation.

## Findings

In this section we first present our findings regarding the HR information, specifically outlining what the HR professionals in the HR department do in terms of communicating in a distinct, consistent, and consensus-oriented way. Following that we elaborate the relationship between line manager beliefs about HR and how it varied depending on the context of these three different units.

### HR information

In terms of providing information about HRM-related matters in the organization, the HR manager and the other professionals in the HR department mainly focused on three activities in their daily work: increasing the internal and external visibility of the HR department and HR practices, achieving consistency and alignment in HR practices across units, and creating consensus about strategic and operational HR with top management of the company. In regard to distinctiveness, visibility emerged as a key focus in the work of the HR department. A clear emphasis on visibility for both HR practices and the HR department itself could be discerned in most interviews. For instance, performance management practices were highly visible in the company throughout the year, with many reminders being sent out to line managers and employees about the time schedule for appraisal meetings and the yearly HR clock. Overall, however, the HR manager seemed to place more emphasis on the visibility of herself and her team as a way of keeping HR issues on the agenda and in everyone’s minds.

*“You are social, you are around, you are not in your office with your door closed. You want to be part of what is happening in each unit, their life. That’s a key.”* (Ruby, HR Manager).

The HR manager also raised the importance of being visible in the different units by visiting regularly, and having knowledge about individual employees, their working reality, their capabilities etc. in order for the HR department to gain credibility and legitimacy of authority in the eyes of line managers, employees and top management. Another central way in which the HR department was visible was the organizing of two bi-annual HR days for line managers. The HR manager oversaw the designing of the agenda for these seminars, which commonly related to a certain HR theme (e.g., the new performance management system, employee wellbeing). The clear sub-purpose of these training days was to draw attention to the HR department and emphasize its importance and role in the organization. The drive for visibility appeared closely coupled with the need to demonstrate legitimacy of authority of HR in the organization, and ability to contribute to the company’s bottom line business by demonstrating an in-depth comprehension of the nature of expert IT work and the core capability of the organization. Related to this, the HR professionals also worked hard to assert their own relevance by providing practical and/or legal guidance, and mental support to line managers in potentially problematic situations with individual subordinates (e.g., alcohol/drug problems, coping at work, lay-offs).

*“Many times they (line managers) tell that “we want “this” to happen.” And then I tell them how we make “this” happen so that we follow all the laws. So that is how this (HR in Tecco) generally works.”* (Lila, HR Business Partner).

The HR manager, as well as the other professionals in the HR department clearly saw the pursuit of consistency and alignment of HR across the organization as one of their key responsibilities. Consistent messaging was a central focus point in the HR department’s work. The HR manager viewed consistency within the HR department team members as a crucial precursor for ensuring they communicated in the same way to line managers, thus aiming at enhancing consistency amongst them regarding the implementation of HR.

The HR professionals also talked about the importance of creating a shared understanding, within the HR department and the organization more widely, about HR goals and practices, in order to improve the validity of their HR practices. There was one joint formal, virtual weekly meeting in which everyone participated, and in addition, many daily, impromptu phone calls and meetings between the HR manager and different HR professionals. These were all measures to ensure continuous consistency of their daily flow of work and HR messaging.

*“We (my team and my boss and I) work together like a many handed giant where each hand knows what they do and don’t get mixed up with the other stuff.”* (Lila, Business Partner).

The HR manager was clearly the driver of the company’s people strategy, operating with firm support from the top management team in which she was also a member. She acknowledged the role of top management support in HR decision-making and mentioned that achieving consensus on important HR matters with them was central in order to carry out her own work. Our interviews with top management echoed this sentiment as they all described the HR manager as a valuable sparring partner regarding people issues and decisions within the organization. While the HR manager and her team were actively working to create consensus with top management, there was no extensive focus on achieving consensus with the line managers. The HR manager clearly viewed it as the role of line managers to implement HR’s vision of people management in Tecco without questioning it or being part of creating it. There was also no visible attempt from HR to work toward creating a deeper internalization amongst the line managers in terms of the purpose or value of HR. Ensuring fairness amongst employees was, however, raised as an important responsibility of HR and mentioned repeatedly in the interviewees with all the HR professionals. The HR professionals’ definition of fairness was treating everybody exactly the same, and a considerable part of HR departments’ time and attention was spent on ensuring that every part of the organization followed the exact same rules and regulations and had the same frames for compensation, benefits, and holidays.

### The interplay of unit context and line manager beliefs about HR

Despite the HR department’s efforts to communicate distinctively and consistently about HR matters and perhaps at least partially due to their eagerness to ensure consensus only with key decision makers (top management), but not with the line managers, there was considerable variability in line managers’ beliefs about HR in our three units. We will now discuss this interplay in more detail, illustrating the interplay of unit context and line manager beliefs in each of our three sample units.

#### Unit CAPTOO

Two factors in the context of the CAPTOO unit stand out as particularly important for illustrating the contextual impact on line manager beliefs about the HR department. The first concerns the nature of the industry in which the unit is operating. The CAPTOO unit is a professional service firm (PSF), characterized by high task complexity, creative and problem-solving work, and an environment where routines and standardized work processes are considered to be of little value (Alvesson et al., 2015). The work force in the unit consists of consultants, a professional group characterized by a strong professional (elite) identity (Alvesson, 2004).

The second contextual factor concerns the organizational structure and decision-making autonomy in the unit. The CAPTOO unit became part of Tecco through an acquisition 4 years ago (at the time of the data collection). Prior to that, the CAPTOO unit had a flat structure, also in terms of HR, which meant that all line managers had considerable discretion about how to handle HR-related matters ranging from recruitment to training and compensation. The CAPTOO unit line managers were thus used to managing the HR processes in their units and perceived the initiatives of corporate HR (for instance concerning recruitments of key people) as interference and meddling with line manager responsibilities. They also did not feel included in the decision making about HR questions to the extent they thought they should be. In addition to all this, the CAPTOO unit is located in Sweden, a country characterized by a national working culture with strong emphasis on consensus-seeking and negotiation in workplaces. We argue that this type of contextual setup, an initially flat structure and strong reliance on negotiations resulted in the unit line managers interpreting HR information and actions as authoritarian and exclusive. The exclusion of line managers from what was previously their own work, the decision-making regarding HR matters cultured their beliefs about the HR department and its abilities.

Previous research (Empson, 2001) has established that wider changes in PSFs, such as those following acquisitions, must be handled carefully so as not to alienate the professionals in the acquired organization. Furthermore, the perceived corporate image of the acquiring company is likely to influence perceptions of employees in the acquired company (Empson, 2001). These factors are visible in our data as well. For example, at the time of our fieldwork a recent Tecco employer branding initiative was considered as a big success by the HR manager and heavily criticized by the line managers in the CAPTOO unit. The line managers described the initiative as too directed toward a male audience and thus having negative implications for the diversity image of the CAPTOO unit and one of its key focus points: competing for scarce consultant talents, an increasing number of which were women. These types of initiatives seemed to further cement the CAPTOO units line managers’ views of their HR department. They described the HR as simply imposing copy paste concepts throughout the company. Consequently, out of all the line managers in our study, those working in the CAPTOO unit clearly held the most critical beliefs about the HR department, its capabilities, and the way in which the HR professionals worked. Their view was that HR is, and should be, a support function with the primary task of servicing the units based on the needs, wishes and orders of the line managers in those units.

*“I think we [the unit line managers]need to be in the driver’s seat, so if they [HR] build more processes like development discussions, a clear dialogue [is needed]; that they actually act as a support function.” (Harry).*

The CAPTOO unit line managers openly questioned HR’s legitimacy and the HR team members’ capabilities to understand the consultancy business context which they perceived to be lacking. The line managers’ questioning of HR’s role, capabilities, and approach to doing HR, was coupled with them speaking favorably of their own HR capabilities and expressing a strong wish to maintain control over their own unit’s HR matters.

*“A bit like hospitals. You should have a doctor heading the hospital in order for the other doctors to accept the person, or the rector of a university should have a Ph.D.” (Stephen).*

*“I guess I think that we need to take care of the strategic HR work ourselves. I don’t think you can at the corporate level, it’s at least very difficult, partly because you need to know a lot about the business, and also the culture in our part of the company, to be, strategically focused on these matters” (Gordon).*

The CAPTOO unit line managers further criticized the HR manager for allowing the HR team to spend too much time on activities that did not significantly contribute to the unit’s bottom-line performance, and for also trying to coerce line managers to do the same. The CAPTOO unit line managers all expressed concern that the HR managers and the HR team’s approach to doing HR in the company, as well as the HR department’s view of its own role did not fit with the context and type of work in their unit.

*“It [the HR department] is a bit stiff and has an old-fashioned attitude at least from our perspective[.] If you’re located in this city and are trying to recruit these high flying IT consultants that we need, you know, they are incredibly talented and very spoiled. They get phone calls from headhunters every week, I promise[.] And I think HR doesn’t really understand our reality. So they’re a bit stiff, like now we’re organizing this training for managers, and please note that attendance is mandatory, in capitals and you’re like wait a minute.*” *(Leslie).*

*“You can’t simply sit in an old support function and then just simply drop things on units and think it can work just like that” (Harry).*

All the CAPTOO unit line managers were of the opinion that HR should involve line managers in decisions related to the implementation of HR activities rather than deciding on their own accord, something they referred to as a *“doing HR for the sake of it”* mentality they perceived as old-fashioned. One example of a decision that had irked many was HR’s decision to introduce mandatory participation in the bi-annual line manager days. In fact, all the line managers were of the opinion that they should be the ones calling the shots regarding HR issues and that the HR department should act as an implementer of their will rather than the other way around which was how the HR team perceived its role, as the “owner” of people related matters in Tecco. To summarize, the CAPTOO unit’s working context where consensus seeking and individual expertise are seen as central values, clashed directly with the HR department’s aims and focus on consistency and full standardization of HR throughout the organization. The CAPTOO unit line managers all seemed to believe that HR’s role should be one of support, with the line managers in charge of strategic people related decisions. They wished for a tailormade HR service at their own demand, and strongly resisted HR practices being imposed on them without their prior involvement and acceptance. The HR information was not interpreted in an intended manner and the result was a clearly lowered motivation to follow or pay attention to the HR’s recommendations and guidelines.

#### Unit NORDSIDE

The context of the NORDSIDE unit includes three key factors that serve to shed light on that unit’s line managers’ beliefs about their HR. First, whereas the CAPTOO unit business was highly knowledge intensive, the work in the NORDSIDE unit concentrated on customer service in a call center, coding, and to a lesser extent providing various IT solutions for customers. The work in this unit had lower task complexity and was generally much more routine driven. This type of more standardized work could also be perceived as more boring or repetitive work, which then may at least partially explain why this unit saw more value in various extra activities and staff events organized by HR.

Second, the NORDSIDE unit was the old headquarters of Tecco, the place where the company was founded. It had the most hierarchical structure of all of the case units in our study. Line managers in this unit were used to managing their subordinates top down, and they were also used to accepting the authority of the HR department, also when they were not included in deciding about HR matters. Finally, and in contrast to the two other units, the NORDSIDE unit is located in a rural area. Previous research (e.g., Culliney, 2017) suggests differences between rural and urban areas in terms of options on the labor market: in general, urban areas have more options and as a consequence mobility and migration is higher in comparison to rural areas. This means that people in rural areas can be expected to be much more dependent on their workplaces and therefore also more accepting of the structure and characteristics of the organization, knowing that they have less opportunities to move to other organizations. This contextual setup may further influence the employee and line manager views of their current positions and at least partially explain why, in stark contrast to the managers in CAPTOO, the line managers in the NORDSIDE unit harbored very positive beliefs about the HR department. The managers viewed HR as working actively and successfully to create a good social environment both in the company and in their own unit, in particular in terms of being an active organizer of various social events such as weekly Friday coffees, as well as Christmas and summer parties. Most notably, however, the NORDSIDE unit line managers voiced appreciation of HR’s efforts in trying to ensure equal and fair treatment of all employees in the organization, and perceived HR to be both active and successful in promoting physical and emotional wellbeing at work and a good organizational culture. Many mentioned HR’s continuous work in closely monitoring employee satisfaction in the company, acting in line with the satisfaction survey results to tackle potential problems and injustices, and continuously encouraging line managers to do the same.

*“It [employee satisfaction] is always discussed that it’s an important goal for us [line managers]. In all information sessions, employee satisfaction and things related to that are on the agenda. You can see that it shows here that it’s professional, there is investment in it and a will that everybody takes it seriously.” (George).*

The NORDSIDE unit line managers further emphasized that HR understood and met their needs as line managers well. They perceived the HR department provided them with both practical and mental support, as well as useful information and help in difficult situations. They used examples such as dealing with subordinates with alcohol-related or psychological problems. They appreciated the existence of formal HR practices and frameworks, such as the performance management system, viewing these as clear and helpful in terms of enhancing their ability to manage with clear cause and effect structures.

*“All the processes have been thought through, from the beginning to the end. For example, bringing in a new user or person into the house. Exiting a person*…*” (Shawna).*

The line managers in the NORDSIDE unit clearly accepted the HR department as an authority on people issues and were happy to work together with them. They emphasized Tecco’s long-standing history of personnel focus and were favorably disposed toward the HR department, viewing them as collaborators and supporters, and repeatedly pointed out that HR was carrying out its role well. In terms of organizational context, the NORDSIDE unit was characterized by an appreciation of clear guidelines and processes related to managing people in the unit. Line managers in this unit considered HR as the clear authority regarding decisions about HRM and did not see their own role in any conflict with this as they considered themselves primarily as implementers of HRs decisions. It is notable that the HR department itself was also characterized by a strong hierarchy, and that the entire HR team shared an appreciation for smooth, clear and formalized processes for the entire organization. In other words there was a good fit between the working context of the NORDSIDE unit and that of the HR department.

#### Unit CAPONE

In the CAPONE unit the contextual factor that stands out as particularly important when trying to understand line managers’ beliefs about HR, is the nature of the expert IT work that constitutes the core capability of the unit. Many CAPONE employees were experts in very specialized areas of IT, making them attractive on the job market and all the more important to retain in the company. The CAPONE unit line managers emphasized the need for HR to really comprehend this, and demanded more realization on part of HR. They argued that as a result of the nature of their business and expertise a much improved level of HR differentiation was needed in terms of salary, holidays, and possibility to allow flexible working hours. They pointed out this was the case particularly in comparison with the NORDSIDE unit which did not face any of these demands in terms of attracting and retaining employees.

The CAPONE line managers further described a discrepancy/clash between the actual office context in the CAPONE unit, and the HR manager’s standardized vision of what the context should be. For example, since many of the IT experts worked on their own, with no customer contact they tended to dress fairly informally. Line managers described how the HR focused on visuals such as dress code whereas they felt the focus should be on competence and achievement. Another example was the CAPONE unit kitchen which had recently been expensively and extensively renovated and equipped with designer furniture and top brand coffee machines. Shortly after, an old water heater had appeared in the kitchen, causing the HR manager to publicly announce this as unacceptable, and potentially detrimental to the company’s visual image since guests too visited the kitchen. The next day an old samovar had appeared anonymously in the kitchen causing considerable mirth among the employees and anger from the part of the HR manager. The line managers raised this in the interviews as an example of how HR’s work in trying to enforce a new company image and implement practices that do not have buy-in and back-up of other organizational members can backfire.

In contrast with the first two units described in this section the CAPONE unit line managers held very mixed beliefs about HR. On the one hand they considered HR to work well in terms of contributing positively to creating a good working environment and maintaining working processes.

*“It [the HR function] defines the rules of the game for employees. It defines the rules of the game for me as a line manager regarding how to treat my subordinates* [.] *Yes, the HR function gives line managers the tools for the daily, or overall work.” (Bert).*

On the other hand, some of the CAPONE unit line managers desired more flexibility from HR in terms of adapting to the needs of different employee groups and a better understanding of the company context and business. Several argued that HR spent too much time on seemingly irrelevant matters (dress code and kitchen etc.) at the expense of other more important issues, such as how to adapt HR practices to better suit the nature of work in the CAPONE unit. This was coupled with general doubt of HR’s understanding of the nature of work carried out by the knowledge workers in the CAPONE unit, and consequently critical comments regarding the relevance of some of the activities of the HR department.

*“It’s nice that we have nice offices and so on, but the problem with HR is that they don’t understand the nature of our work, what expert work is. For example, when they planned these premises, HR’s view was that ‘why on earth do you need big tables, you can enlarge things on laptops’ and so on. And an expert has three screens and about one hundred apps open. Experts work in a completely different way than someone who sits somewhere with a laptop. They [the experts] need peace and quiet around them and certain working tools. We have people over there who have ticketing machines at their work desks. So those don’t really fit on a 60 cm wide table and then you just pick them up and carry them around. A 100 kg machine.” (Natasha).*

As opposed to their colleagues in the NORDSIDE unit and more in line with those in the CAPTOO unit, the line managers in the CAPONE unit found that the HR department was lacking in flexibility and understanding of how to adapt to different situations. They considered the HR department as being overly focused on following guidelines and processes at all times just for the sake of it, and not being able or willing to assess what makes sense and when.

“… *they [HR] didn’t understand, for instance, that if we are employing a top expert [.] then they do everything by the book, “your first holidays will be in 2 years.” If you take a grown man, who is a top-notch expert, I don’t think we need to negotiate about these things with HR. They should already understand better beforehand that they should have a [better] “eye for the game”. The HR manager does. She is, after all, an older, experienced woman. As for the others [in the HR team], this is not necessarily the case. And I wouldn’t always want to have to go to the HR manager straight away. They’re in a way prisoners of their own rules.” (Bert).*

To summarize, the unit context of the CAPONE unit can be characterized as a mix between the CAPTOO unit and the NORDSIDE unit contexts. On the one hand the unit also identifies as doing expert work (CAPTOO) and simultaneously the unit context is close to the clear hierarchies of the original headquarters of Tecco (NORDSIDE). Line managers in the CAPONE unit seemed to recognize HR’s efforts in trying to achieve alignment throughout the organization but they also felt that HR’s ability to provide sufficient and relevant support was limited by their lack of knowledge about the nature of expert work. The line managers in the CAPONE unit emphasized the lack of capabilities of HR in terms of fully understanding the business and expertise of the unit and believed that this hindered the HR from providing adequate support. Thus, they too were interpreting the HR actions from the perspective of contextual clash.

## Discussion and conclusion

### Theoretical implications

A majority of research on HR attributions has focused on explaining how HR attributions are related to various individual and organizational outcomes (e.g., Van De Voorde and Beijer, 2015; Shantz et al., 2016; Hewett, 2021; Sanders, 2022), but more recently there has been an increased interest in examining the formation of HR attributions (Hewett et al., 2019; Wang et al., 2020; Alfes et al., 2021). Kelley and Michela (1980) suggest information, beliefs, and motivation shape attributions and call for more research into the interplay between these elements. This paper contributes to our understanding of how line managers’ beliefs regarding the HR department depend on the information they receive from HR as well as the context in which they work.

In terms of information from the HR department, our case organization’s HR department spent a significant amount of their working time ensuring a distinct and consistent (fair) HR system for the entire organization, in line with what Bowen and Ostroff (2004) suggested is important for building a strong HRM system. The HR department also argued that consensus with top management on HR matters, but did not give any strategic significance, or spend time working to create consensus with the line managers of the organization. Our findings illustrate that one clear message can be, and in the case of our sample units was, interpreted in three very different ways. In our case, this largely depended on the unit context in which the line managers are employed, where the characteristics of the unit, it’s age, history, nature of business, unit culture among other things, served to shape line manager beliefs about the role and purpose of the HR department, as well as their own HR responsibilities. This extends our understanding of the reasons that underlie variability of line manager beliefs of HR within organizations, which recently (e.g., Van Rossenberg, 2021; Meier-Barthold et al., 2022) has been suggested as an important next step for further increasing our understanding of HR attributions and the microprocesses through which they are formed. It also contributes to existing research on HRM system strength and HR attributions by highlighting the importance of focusing not only on the consistency of the HRM system, but also on individual line managers beliefs about HR, as well as on the possible match/mismatch with the context in which the HR processes take place.

Our research also contributes to recent calls for increasing our understanding of the role of social context for attribution formation (e.g., Beijer et al., 2019). The central contextual factors influencing line manager beliefs about HR in our research are the history of the unit in terms of hierarchies and the working processes. Nature of work conducted also plays a role as experts and professionals have a tendency to see themselves as capable of taking care of their unit’s people issues better than the HR department does. Further, where work duties are more repetitive, HR initiatives such as creating more fun at work are more appreciated. Finally, in our research, the unit with a mix of expert and more repetitive work, the reception of the HR department was more mixed, with some of the line managers appreciating the work from the HR department and its activities more while others line managers appreciate this less, questioning the HR capabilities (more of an expert role). The expert workers would also have preferred more consensus, they would have liked the HR department to work on creating consensus on how things are done with them (not only top management), and when this did not happen their motivation to interpret HR information in the intended way further decreased.

Finally, in addition to increasing our understanding of the interplay between line managers’ beliefs regarding the HR department, the information they receive from the HR department, and their motivation conceptualized as unit context, our study contributes to understanding this process from different levels. While previous research mainly focuses on the individual level (Kelley and Michela, 1980; Hewett et al., 2019), our approach includes antecedents at the individual (line manager beliefs), organizational (information from the corporate HR department) and unit levels (unit context) to understand the complex interplay of these antecedents at different levels leads to variability of HRM, and also affects the way the HR information is received. Our study findings also underscore the importance of the context for the formation of attributions formation on a more general level, pointing to the need for a deeper and wider investigation of different kinds of contexts e.g., team/organizational/occupational/national, high/low HR devolvement, good/poor HR reputation and how these shape HR attributions in potentially different ways. Thus, our findings serve to deepen our understanding of the dynamic microprocesses through which HR practices do or do not influence performance (Hewett et al., 2018), namely, the complex interplay of elements at different levels that then in turn shapes individual attribution formation.

### Practical implications

The findings of this study show that line managers of three units of a Nordic company in the IT sector responded differently to similar information from the HR department. While it is important to ensure that the corporate HRM system can be characterized by distinctiveness, consistency, and consensus, our findings suggest it is not sufficient, and that attention also needs to be directed at line-managers’ beliefs and the context in which HR processes take place. This means that in addition to the importance of sending out distinctive, consistent and consensual information to line managers, organizations, especially senior managers and HR professionals need to be aware that this is not sufficient, and that in order to get the message across the receivers of the information and the context they are in matter for the outcome. This means that for HR’s signaling success, the relative importance of these elements may differ depending on the line-managers’ motivational context and beliefs about the HR department. This also means that despite the importance of consistency, differential treatment of different units should be considered, depending on the characteristics of the specific unit.

When it comes to line managers, they differ from employees in the sense that they are actively involved in carrying out and implementing many HR initiatives. The HR department should take into account that while distinctiveness may be central for employees (knowing how to fill out the performance appraisal form and when to send it in), HR may need to focus more on consensus seeking when sending out information to line managers. Seeking consensus about HR issues between the HR department and the line managers of specific units is key for maintaining the legitimacy and authority of the HR department, and similarly consensus in terms of ensuring line manager buy-in into the HR initiatives being signaled may be crucial for ensuring subsequent effective information sending onward to employees. Furthermore, there may be a need to adapt the information sending style to managers in different units depending on contextual factors. For example, managers in flat hierarchies may desire more consensus-driven signaling as their feelings of fairness to be included in the decision-making from the line-managers have an influence on their willingness to pick up signals from the HR department. The HR department should be aware of these effects and act accordingly.

### Limitations and future research directions

Just like other studies, this study is subject to certain limitations. First, although our study highlights the role of context for the forming of HR attributions, one could argue that an even more detailed and rounded perspective of context is needed. In addition, more and other contexts should be considered. We acknowledge this as a limitation of our study and consider our study a first step in this direction, calling for future work, both qualitative and quantitative, that considers the environment in different organizational and national contexts. For instance, organizations in different industries, such as health care and education, and countries differing in cultural values should be considered in future research.

Second, in this study we focused on line managers as key mediators between the HR department and employees, but did not take senior management and employees into account. Therefore, we encourage future studies to include a focus also on actors who intend to implement new HR policies (senior managers) and the end receivers, namely, the employees. Multi-actor and multi-method studies, quantitative methods paired with qualitative that include HR, senior and line managers, and employees to consider the trickle-down processes of information from the HR department hold considerable potential to understand the whole process from intended HR practices to employee and organizational performance. Our analysis process would have also been even more thorough had we been able to combine our qualitative analysis with quantitative data. Approaching this kind of complex issue with multiple methods would add even more depth to the analysis process. Another essential step for HR attributions research going forward is to strive for increasingly contextualized contributions that enable more fine-grained examinations of HR attributions in specific organizational contexts.

## Data availability statement

The datasets presented in this article are not readily available because participants have not agreed to sharing data with others. Requests to access the datasets should be directed to jennie.sumelius@hanken.fi and hertta.vuorenmaa@aalto.fi.

## Ethics statement

Ethical review and approval was not required for the study on human participants in accordance with the local legislation and institutional requirements. The patients/participants provided their written informed consent to participate in this study.

## Author contributions

HV and JS contributed to the design of the study, collected the data used, conducted the data analysis, wrote the first version of the findings section of the manuscript, and contributed to the manuscript revision. KS wrote the first draft of the theoretical background and later contributed to the further developing the findings section. All authors read and approved the submitted version and contributed to writing the introduction of the manuscript.
